# Methicillin-Resistant *Staphylococcus aureus* (MRSA) in Slaughter Houses and Meat Shops in Capital Territory of Pakistan During 2018–2019

**DOI:** 10.3389/fmicb.2020.577707

**Published:** 2020-09-28

**Authors:** Asma Sadiq, Maroof Samad, Saddam, Nosheen Basharat, Shahid Ali, Roohullah, Zubaida Saad, Allah Nawaz Khan, Yasin Ahmad, Alam Khan, Jadoon Khan

**Affiliations:** ^1^Molecular Virology Laboratory, Department of Biosciences, COMSATS University Islamabad, Islamabad, Pakistan; ^2^COMWAVE Institute Islamabad, Islamabad, Pakistan; ^3^Pakistan Institute of Medical Sciences (PIMS) Islamabad, Islamabad, Pakistan; ^4^Department of Microbiology, Abdul Wali Khan University Mardan, Mardan, Pakistan; ^5^Department of Microbiology, Quaid-i-Azam University (QAU), Islamabad, Pakistan; ^6^Department of Zoology, Women University Mardan, Mardan, Pakistan; ^7^Department of Life Sciences, Abasyn University, Islamabad, Pakistan

**Keywords:** LA-MRSA, phenotypic, antibiotic sensitivity, PCR, *mecA* gene 3

## Abstract

Livestock-associated methicillin-resistant *Staphylococcus aureus* (LA-MRSA) is a major concern in many parts of the world, including Pakistan. The aim of this study was to investigate the prevalence of MRSA in slaughterhouses and meat shops in Rawalpindi-Islamabad, Pakistan, 2018–2019. A total of 300 samples were collected: 40 from each of working area, tools (knives, hooks), butcher hands and beef, 30 from each of chicken and mutton, 20 from each of nasal and rectal swabs. *S. aureus* was phenotypically identified by performing gram staining and biochemical tests. 150 of the 300 samples were confirmed to be *S. aureus* by phenotypic identification. MRSA was identified among *S. aureus* positive isolates by performing disk diffusion test and by detecting *S. aureus*-specific genes such as 16s rRNA, *nuc*, *mecA*, *spa*, and *coa*. Out of 150 isolates 96 (63%) showed resistance to antibiotic cefoxitin, known as a potential marker for detecting MRSA. While all 150 isolates have shown complete resistance to the four antibiotics neomycin, methicillin, ciprofloxacin and tetracycline. The *nuc* and 16s rRNA genes were detected in all 150 *S. aureus-*positive isolates and 118 (79%) were confirmed to be MRSA through the detection of the *mecA* gene. MRSA prevalence was highest in chicken (23/30, 77%) followed by beef (25/40, 63%), mutton (15/30, 50%), knives (18/40, 45%), nasal swabs (7/20, 35%), working area (11/40, 28%), rectal swabs (5/20, 25%), hooks (7/40, 18%), and butcher hands (7/40, 18%). 50 MRSA-positive isolates were chosen to identify two virulence factors (*spa* and *coa* gene). Of the 50 MRSA isolates subject to *coa* and *spa* gene typing, 27 (54%) were positive for the *coa* gene and 18 (36%) were positive for the *spa* gene, respectively. To the best of our knowledge, this was the first study on the molecular identification of MRSA in meat samples from Pakistan. High prevalence of MRSA in meat samples demand for implementation of proper hygienic practices and procedures during the slaughtering, transport and marketing of meat and meat products in order to prevent the spread of these bacteria to the human population.

## Introduction

*Staphylococcus aureus* is a Gram-positive, coagulase- positive pathogen belong to the family *Staphylococcaceae*. This is a spherical bacterium about 1 μm in diameter that forms grape-clusters (Lakhundi and Zhang, 2018). *Staphylococcus aureus* (*S. aureus*) is one of the most common microorganisms colonizing the nasal cavity of humans and different animal species (Al-Amery et al., 2019). This may also be found in external body surfaces as either commensal or pathogenic bacteria that can cause multiple infectious diseases (Weese and van Duijkeren, 2010). *S. aureus* has a variety of virulence factors and toxins, frequently responsible for many toxin-diseases including toxic shock syndrome, staphylococcal foodborne diseases (SFDs), and scalded skin syndrome (Okoli et al., 2018). *Staphylococcus* has the potential to establish resistance to broad-spectrum antibiotics in a short period, such as the β-lactam group of antibiotics, aminoglycosides and quinolones commonly used in clinical practice for the treatment of serious infections (Deurenberg et al., 2007). Methicillin-resistant *staphylococcus* (MRSA) strain was first identified in United Kingdom in 1961, continues to be a serious hospital concern for public health (Patricia Jevons, 1961; Lowy, 1998). The United States Centers for Disease Control and Prevention (CDC) reported in July 2002 the first *S. aureus* strain resistant to both vancomycin and methicillin (Graveland et al., 2011). Nonetheless, unusual strains appeared starting in the 1980s, leading to a global spread.

A distinct MRSA lineage, Clonal Complex (CC) 398, was first identified from pigs in The Netherlands and France in food producing animals in Europe, particularly in pig herds, turkeys, veal calves and broiler flocks (Vossenkuhl et al., 2014). The term “Livestock-associated MRSA” (LA-MRSA) has therefore been introduced, considering that livestock form a new and separate reservoir for MRSA (Larsen et al., 2012). In Asian countries, however, the ST9 sequence, a separate genetic lineage, is predominant among MRSA isolates from livestock (Güven Gökmen et al., 2018). Notably, outbreaks of LA-MRSA in hospitals and invasive infections of LA-MRSA in humans are growing (Lewis et al., 2008). Accordingly, LA-MRSA has become an important public health problem that needs close monitoring.

MRSA strains are resistant to all β-lactam antimicrobials by a penicillin-binding protein (PBP2a) that has a weak affinity to all β-lactams. The protein is encoded by the *mecA* gene, which resides on the lactams of a mobile genetic element called the staphylococcal chromosome cassette (SCCmec) (Ivbule et al., 2017). To classify the epidemiological characteristics of MRSA strains and, more importantly, to research the evolution and spread of disease clones, it is appropriate to employ relevant and reproducibility molecular methods with ample discriminative capacity to track changes in time. Most of the methods used for this are pulsed-gel electrophoresis (PFGE), Multilocus Sequence Typing (MLST), staphylococcal cassette chromosome mec (SCCmec), and staphylococcal protein A (*spa*) and staphylococcal *coa* (coagulase) gene (Cookson et al., 2007). Virulence factors of *S. aureus*, *coa*, and *spa* genes, respectively has been shown to be directly linked to pathogenesis and the magnitude of infection (Rathore et al., 2012). Both virulent genes are highly polymorphic and can provide critical information on strain variations.

The widespread use of antimicrobials in animal production is believed to promote the emergence and spread of MRSA because of selection pressure induced by antimicrobials (Pires et al., 2009). Overcrowding in animal husbandry and intensive animal trade can help the rapid spread of MRSA among the farm animals (Guo et al., 2018). LA-MRSA strains were also found on wholesale in raw meat including poultry, beef, veal, and pork (Alt et al., 2011). Recent studies have also found that LA-MRSA may colonize in multiple animals and associated workers (Rinsky et al., 2013). It indicates possible transmission of cross-contamination in the chain during slaughter and processing (Waters et al., 2011). Nevertheless, the scale of this transmission is not well-understood up to now.

Pakistan, as a developing country, suffers primarily from antibiotic resistance, which is a concern not only for Pakistan, but for the entire human/animal population (Ali et al., 2018). Weak steps to monitor infections as well as continued unregulated exposure of humans and animals to antibiotics have contributed to this enormous problem of MRSA development and transmission (Lakhundi and Zhang, 2018). This in effect restricts the treatment options for MRSA infections. Continuous monitoring for MRSA is therefore necessary in any setting by analyzing the characteristics, host specificity, and propagation paths of newer strains.

Detection of staphylococci in meat is often related to poor hygienic practices during processing, shipping, slicing, storage and point of sale by individuals involved in the production process. Therefore, the main objective of this study was to identify *S. aureus* phenotypically and molecularly through amplification of 16S ribosomal RNA, *mecA*, *nuc*, *spa*, and *coa* genes from selected slaughter houses and meat shops in twin cities Rawalpindi, Islamabad, Pakistan during 2018–2019.

## Materials and Methods

### Ethical Approval

The ethical approval of the study was taken from ethical review committee of Sarhad University, Peshawar and Pakistan Institute of Medical Sciences (PIMS), Islamabad, Pakistan. The institutional committees approved the experiments carried out for the current research described in the “Materials and Methods” section of the manuscript. All procedures have been carried out in compliance with the relevant regulations and standards.

### Sample Collection and Processing

Three hundred samples from various slaughterhouses and meat stores in different areas of Rawalpindi, Islamabad, Pakistan were collected during 2018–2019. The sample size for this study was calculated using prevalence formula in a software N-Query Advisory (STATCON Gmbh, Germany, Version 7.0) (Pourhoseingholi et al., 2013). A total of 300 samples were collected: 40 from each of working area, tools (knives, hooks), butcher hands and beef, 30 from each of chicken and mutton, 20 from each of nasal and rectal swabs. Sterile cotton swabs were first dipped in buffer peptone water (Oxoid, United Kingdom) rubbed horizontally and then vertically on the selected materials (Adugna et al., 2013). Then swabs were placed with proper labeling in airtight zip bags and stored at −20°C. Collected samples were processed through standard operating procedures. The raw meat samples (1 g) were first added to 10 ml tryptone water (10 g/l tryptone and 5 g/l NaCl) and properly mixed. This solution was used as inoculum for making serial dilutions. This makes the first dilution i.e., 10^–1^. The dilutions 10^–3^, 10^–4^, and 10^–5^ were used for inoculation. The swab samples were placed in peptone water-containing falcon tubes and then centrifuged at 5,000 rpm until all material on swab is dissolved fully in peptone water.

### Isolation and Phenotypic Identification of *S. aureus*

Samples were initially grown on selective medium mannitol salt agar (MSA) for the growth of *S. aureus* using cotton swabs. A sample of 100 μl was transferred to the Mannitol salt agar (Thermo Fisher Scientific, United States) medium and distributed uniformly. The plates were incubated at 37°C overnight. Isolated colonies that showed fermentation on MSA medium were subcultured again on the MSA in order to get pure cultures. Bacterial isolates were cultured on blood agar (Sigma-Aldrich, Germany) medium containing 5% heparin free sheep blood added to the blood agar base after autoclaving and cooling to 50°C. *S. aureus* have the ability to hemolyse sheep blood and shows alpha (α) hemolysis observed in the form of clear zones around colonies. Until observations, plates were incubated at 37°C for 24–48 h. *Staphylococci* have been detected phenotypically using standard methods specific to the detection of enzymes and certain biochemical processes. These techniques include culturing, gram staining, catalase test, coagulase and DNase test (O’Brien et al., 2012). Following the phenotypic study of *S. aureus*, isolates were stored in a nutrient broth with an addition of 20% glycerol to prevent any frost shock due to crystal formation in bacterial cells.

### Antimicrobials Susceptibility Test

To confirm the antibiotic susceptibility of bacterial isolates to specific antibiotics, Kirby Bauer or disk diffusion test was used (Bauer et al., 1996). Mueller Hinton agar medium (MHA) was used for disk diffusion test (Oxoid Ltd., United Kingdom). The antibiotic disks that were selected for the antibiotic susceptibility test were (novobiocin 5 μg, cefoxitin 30 μg, neomycin 30 μg, methiciline 5 μg, amoxycilin 30 μg, erythromycin 15 μg, gentamycin 5 μg, vancomycin 30 μg, ciprofloxacin 5 μg, and tetracycline 30 μg), respectively. These disks were carefully placed on the Petri dishes to avoid any environmental contamination. The results were noted after overnight incubation at 37°C according to the Clinical and Laboratory Standards Institute (Clinical and Laboratory Standards Institute [CLSI], 2019).

### PCR Detection of 16s rRNA, *nuc*, and *mecA* genes of *S. aureus*

A molecular analysis of phenotypically identified *S. aureus* isolates was performed by amplification of the *Staphylococci* 16s rRNA gene, the species-specific *nuc* gene and the *mecA*-resistant gene. The genomic DNA was extracted using the DNA extraction method CTAB (Cetyltrimethylammonium bromide) (Maristela Oliveira Lara et al., 2018). Before molecular analysis, the extracted DNA was analyzed using gel electrophoresis (1%). Molecular typing was carried out for one MRSA-like colony per positive sample. The total volume of reaction mixture was 20 μl that contained 1 μl each of forward and reverse primer, 10 μl of Wizepure 2X PCR master mix (Wiz biosolutions, South Korea), 7 μl of molecular grade water and 1 μl of template DNA. Gene specific primers were used as described by Murakami et al. (1991) and Salisbury et al. (1997) (Table 1). The PCR conditions set for16s rRNA and *mecA* gene amplification reaction were, an initial denaturation at 95°C for 5 min, 29 cycles of amplification (denaturation at 95°C for 30 s, annealing at 55°C for 30 s, extension at 72°C for 40 s and final extension at 72°C for 2 min). The PCR conditions set for *nuc* gene amplification reaction were, an initial denaturation 95°C for 5 min, 34 cycles of amplification (denaturation at 95°C for 30 s, annealing at 53°C for 30 s, extension 72°C for 30 s, final extension 72°C for 2 min). For product visualization, 8 μl of DNA sample was mixed with 2 μl of 6× loading dye. Then 1.5% of gel was prepared in 1× TAE buffer and 100-bp DNA ladder (Bioron, Cat. No. 304105, Germany) with a voltage of 100V for 20–25 min was used to validate the amplified product. After the gel electrophoresis was completed, the gel results were visualized using the Gel doc system (Thomas scientific, United States).

**TABLE 1:** List of primers used and reaction conditions for each gene amplified in this study.

**List of primers**			
Primer name	Sequence (5′-3′)	Product size (bp)	References
16S-F	5′-GTGCCAGCAGCCGCGGTAA-3′	876	Salisbury et al., 1997
16S-R	5′-AGACCCGGGAACGTATTCAC-3′		
mecA-F	5′-AAA ATC GAT GGT AAA GGT TGG C-3′	533	Murakami et al., 1991
mecA-R	5′AGT TCT GCA GTA CCG GAT TTG C3′		
nuc-F	5′-GCG ATT GAT GGT GAT ACG GTT-3′	270	Murakami et al., 1991
nuc-R	5′-AGC CAA GCC TTG ACG AAC TAA AGC-3′		
coa-F	5′-ATAGAGATGCTGGTACAGG-3′	680, 891	Hookey et al., 1998
coa-R	5′-GCTTCCGATTGTTCGATGC-3′		
*spa*-F (x-region)	5′-CAAGCACCAAAAGAGGAA-3′	100, 200	Bhati et al., 2016
*spa*-R (x-region)	5′CACCAGGTTTAACGACAT3′		

### *coa* and *spa* Genes Typing of MRSA

The amplification of *coa* (coagulase) and x region of *Staphylococcus* protein A (*spa*) genes is carried out for 50 selected MRSA isolates based on the identification of *mecA* (resistant) gene. DNA extraction was performed using the QIAamp DNA Mini Kit (Qiagen, Germany), with manufacturer instructions. Gene-specific primers were used as described (Hookey et al., 1998; Bhati et al., 2016). The sequences of primers for *coa* and *spa* genes are given in Table 1. The total volume of the reaction mixture was 20 μl, including the PCR Master mix (Thermo Fisher Scientific, United States), distilled water, 2 μl of the DNA sample and 2 μl of each of forward and reverse primer. The PCR cycling conditions used for *coa* gene were: an initial denaturation at 94°C for 45 s, 30 cycles of amplification (denaturation at 94°C for 20 s, annealing at 57°C for 15 s, extension at 70°C for 15 s and final extension at 75°C for 2 min). The PCR cycling conditions used for *spa* gene were: 34 cycles of amplification (denaturation at 94°C for 60 s, annealing at 55°C for 60 s, extension at 70°C for 60 s and final extension at 72°C for 5 min). For product visualization, 8 μl of DNA sample was mixed with 2 μl of 6× loading dye. Then 1.2% of gel was prepared in 1× TAE buffer having ethidium bromide (0.5 μg/ml) and 100-bp DNA ladder (Bioron, Cat. No. 304105, Germany) was used to validate the amplified product. The voltage used was 80 V for 1 h. After the gel electrophoresis was completed, the results of the gel were visualized using the Gel doc system (Thomas scientific, United States).

## Results

### Phenotypic Identification Results

A total of 300 samples were grown in the mannitol salt agar (MSA) medium. Of the 300 samples, 150 fermented mannitol and showed yellow colonies on MSA (Supplementary Figure S1). In order to obtain pure colonies, *S. aureus* positive samples were subcultured further on MSA and selected for other biochemical tests. All 150 samples were grown in the blood agar, and all showed beta-hemolysis activity (Supplementary Figure S2). Gram staining was carried out for all 150 isolates. Cluster of cocci, purple in color showed a definite pattern under the light microscope at the 100× lens (Supplementary Figure S3). Further catalase, coagulase and DNase tests were conducted on all 150 isolates. All of the tests showed positive results for *S. aureus* (Supplementary Figures S4–S6).

### Prevalence of *S. aureus* Based on Phenotypic Identification Results

The prevalence of *S. aureus* differed among sample sources and specimen types based on the phenotypic findings. Out of a total of 300 samples, 150 samples were positive for *S. aureus* with an overall prevalence of 50%. The highest prevalence of *S. aureus* was observed for chicken (25/30, 83%) followed by beef (30/40, 75%), knives (22/40, 55%), mutton (15/30, 50%), nasal swabs (9/20, 45%), and working area (16/40, 40%). The least prevalence of *S. aureus* was found in rectal swabs (7/20, 35%), hooks (14/40, 35%), and butcher hands (12/40, 30%), respectively (Table 2).

**TABLE 2:** The prevalence of *S. aureus* among 300 samples from different sources.

Isolate ID	Source	Total number of samples (n)	*S. aureus* positivity rate n (%)
A	Knives	40	22 (55%)
B	Hooks	40	14 (35%)
C	Working Area	40	16 (40%)
D	Butcher hands	40	12 (30%)
E	Nasal swab	20	9 (45%)
F	Rectal swab	20	7 (35%)
G	Chicken	30	25 (83%)
H	Mutton	30	15 (50%)
I	Beef	40	30 (75%)

### Antimicrobial Resistance Results

One hundred and fifty *S. aureus* isolates were screened for different antimicrobials using a disk diffusion technique. Table 3 shows the percentage of resistant, intermediate and susceptible isolates for 10 different antibiotic disks. The isolates were completely resistance to the neomycin, methicillin, ciprofloxacin and tetracycline and showed 63 and 52% resistance to cefoxitin and novobiocin, respectively. It was noted that 70, 76, 82, and 80% of the strains were also resistant to amoxycillin, erythromycin, gentamycin and vancomycin, respectively (Supplementary Figure S7). The antibiotic susceptibility and intermediate susceptibility trend differed among 10 drugs. All isolates showed 20, 12, 10, and 8% susceptibility to cefoxitin, novobiocin, amoxicillin and Erythromycin, respectively. While, gentamycin and vancomycin showed equal (6%) susceptibility. It was also observed that 36, 17, 20, 16, 12, and 14% of the isolates showed intermediate susceptibility toward novobiocin, cefoxitin, amoxicillin, erythromycin, gentamycin, and vancomycin, respectively.

**TABLE 3:** Susceptibility to antimicrobials among 150 isolates of *S. aureus*.

S. No.	Antimicrobial agent	Disk content (μg)	Susceptible no. of isolates (%)	Resistant no. of isolates (%)	Intermediate no. of isolates (%)
1	Novobiocin	5	18 (12%)	78 (52%)	54 (36%)
2	Cefoxitin	30	30 (20%)	95 (63%)	25 (17%)
3	Neomycin	30	0	150 (100%)	0
4	Methicillin	5	0	150 (100%)	0
5	Amoxicillin	30	15 (10%)	105 (70%)	30 (20%)
6	Erythromycin	15	12 (8%)	114 (76%)	24 (16%)
7	Gentamycin	5	9 (6%)	123 (82%)	18 (12%)
8	Vancomycin	30	9 (6%)	120 (80%)	21 (14%)
9	Ciprofloxacin	5	0	150 (100%)	0
10	Tetracycline	30	0	150 (100%)	0

### Distribution of MRSA via Molecular Analysis

**FIGURE 1:**
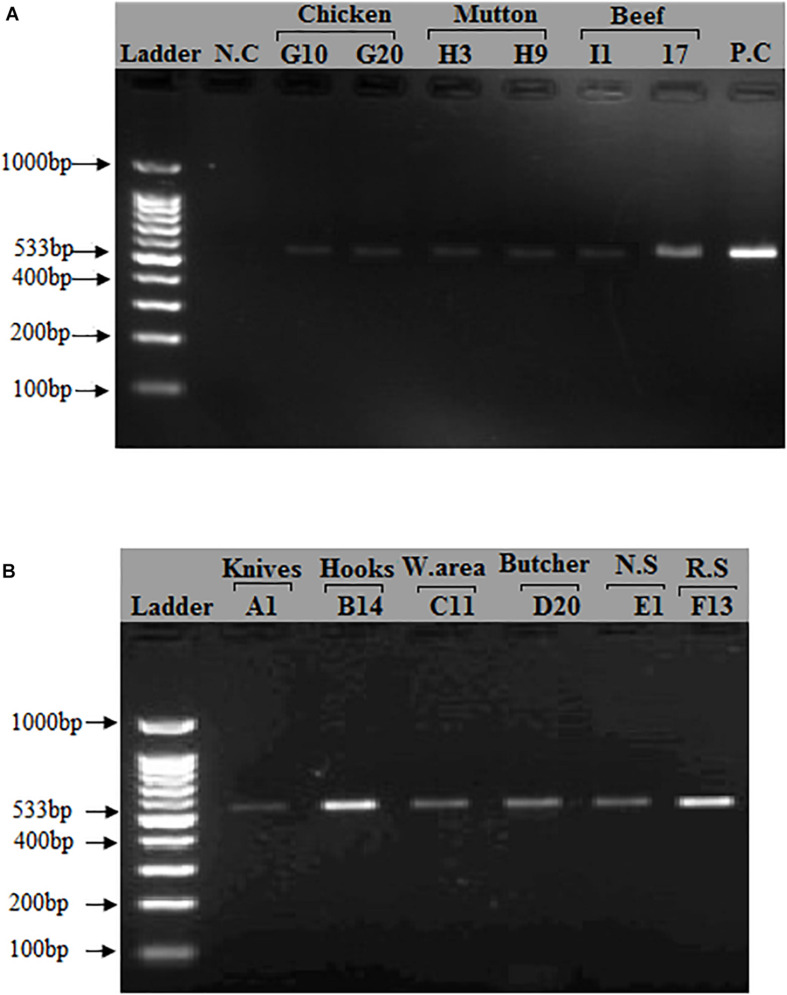
Detection of mecA gene in *S. aureus* isolates: A band (533 bp) corresponding to amplified region of mecA gene. (A) First lane = 100 bp ladder, 2nd Lane = Negative control, Lane# 9 = Positive control (*S. aureus* ATCC 33591), Lane# 3–8 = PCR products of mecA gene from meat samples (Beef, Chicken, Mutton). (B) First lane = 100 bp ladder, Lane# 2–7 = PCR products of mecA gene from different sources (Knives, Hooks, Working area, Butcher hands, Nasal swabs, Rectal swabs, Beef, Chicken, and Mutton).

After phenotypic validation, all 150 isolates undergo molecular characterization to detect methicillin resistant *S. aureus* on the basis of 16S rRNA, *nuc*, *mecA*, *coa*, and *spa* genes amplification. The extracted DNA was run on agarose gel 1% and results were observed on gel doc (Supplementary Figure S8). All 150 *S. aureus* positive isolates were subjected to 16s rRNA, *nuc* gene and *mecA* typing. All isolates were tested positive for 16S rRNA gene and *nuc* genes (Supplementary Figures S9, S10). The *nuc* gene primer gave the PCR product equal to 270 bp. The 16S rRNA primers gave PCR products equal to 886 bp. The methicillin resistant *S. aureus* (MRSA) gene (*mec A*) was identified in (118/150, 79%) of the samples. The *mecA* gene primer gave the PCR product equal to 533 bp (Figure 1). The distribution of MRSA based on the detection of *mecA* and cefoxitin resistance pattern with respect to the specimen type is shown in Table 4. The highest prevalence of MRSA was detected in chicken (23/30, 77%) followed by beef (25/40, 63%), mutton (15/30, 50%), knives (18/40, 45%), nasal swabs (7/20, 35%), working area (11/40, 28%), rectal swabs (5/20, 25%), hooks (7/40, 18%), and butcher hands (7/40, 18%). 95 of the 150 isolates were resistant to cefoxitin and the *mecA* gene was found in all cefoxitin-resistant isolates. 30 out of 150 isolates were susceptible to cefoxitin. 23 out of 30 susceptible isolates showed presence of *mecA* gene (Table 4).

**TABLE 4:** Comparison between *mecA* gene PCR results and cefoxitin disk diffusion test among 150 *S. aureus* positive isolates from different sources.

Isolate ID	Source	Total number of *S. aureus* positive isolates (n)	Cefoxitin resistant MRSA n (%)	MRSA positivity rate (*mecA* gene) n (%)
A	Knives	22	13 (33%)	18 (45%)
B	Hooks	14	7 (18%)	7 (18%)
C	Working Area	16	10 (25%)	11 (28%)
D	Butcher hands	12	5 (13%)	7 (18%)
E	Nasal swab	9	2 (10%)	7 (35%)
F	Rectal swab	7	2 (10%)	5 (25%)
G	Chicken	25	19 (63%)	23 (77%)
H	Mutton	15	12 (40%)	15 (50%)
I	Beef	30	25 (63%)	25 (63%)

### Results of *coa* and *spa* Genes Typing of MRSA

**FIGURE 2:**
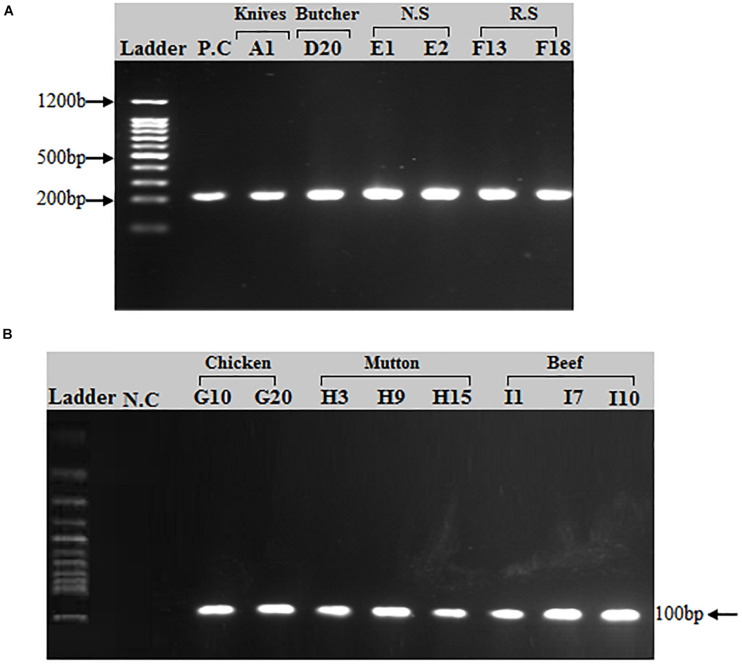
PCR amplification of *spa* (*spa-*X) gene in *S. aureus* isolates: **(A)** A band (200 bp) corresponding to amplified region of *spa* gene. **(A)** First lane = 100 bp ladder, 2nd Lane = Positive control (*spa* gene), Lane# 3–8 = PCR products of *spa* X region from different sources (Knives, Butcher hands, Nasal swabs, Rectal swabs). **(B)** A band (100 bp) corresponding to amplified region of *spa* gene (X region). First lane = 100 bp ladder, 2nd Lane = Negative control, Lane# 3–10 = PCR products of *spa* X region from meat samples (beef, chicken, and mutton).

**FIGURE 3:**
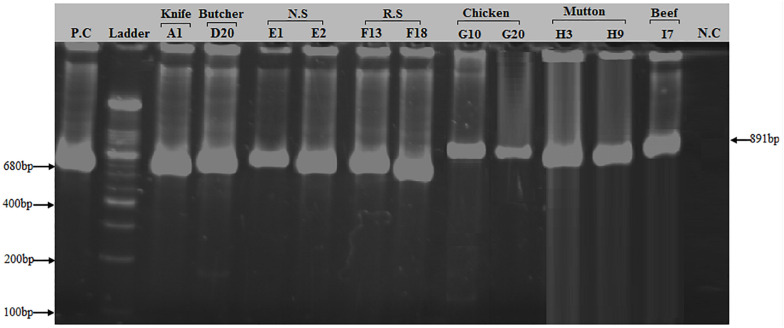
PCR amplification of *coa* (Coagulase) gene in *S. aureus* isolates: Two bands of 680 and 891 bp corresponding to amplified region of *coa* gene. First lane = Positive control (*coa* gene), 2nd Lane = 100 bp ladder, Lane# 3–8 = PCR products of 680 bp size of *coa* gene from different sources (Knife, Butcher hands, Nasal swabs, Rectal swabs), Lane# 9–13 = PCR products of 891 bp size of *coa* gene from meat samples (beef, chicken, and mutton), Lane# 14 = Negative control.

Of the 50 MRSA isolates subject to *coa* and *spa* gene typing, 27 were positive for the *coa* gene and 18 were positive for the *spa* gene, respectively. PCR amplification of the X region of the *spa* gene generated a single amplicon in each isolate. Two amplicons of different sizes (100 and 200 bp) were produced (Figure 2). One hundred bp was more common pattern in isolates numbers G3, G10, G15, G20, G23, H3, H9, H15, I1, I7, I10 and I13, respectively. While, 200 bp was more common pattern in isolate numbers A1, D20, E1, E2, F13 and F18, respectively (Table 5). PCR amplification of the *coa* gene yielded a single amplicon in each isolate. Two amplicons of different sizes (681 and 891 bp) were developed (Figure 3). Six hundred eighty bp was more common pattern in isolates numbers G1, G3, G10, G15, G20, G23, G27, H3, H9, H15, I1, I7, I10, I13 and I17, respectively. While, 891 bp was more common pattern in isolate numbers A1, A13, A35, D9, D20, E1, E2, E11, E14, F13, F18 and F20, respectively (Table 5). The distribution of *spa* gene in different sample types is as follows: knives (1/5, 20%), hooks (0/5, 0%), working Area (0/5, 0%), butcher hands (1/5, 20%), nasal swabs (2/5, 40%), rectal swabs (2/5, 40%), chicken (5/8, 63%), mutton (3/5, 60%), and beef (4/7, 57%). The distribution of *coa* gene in different sample types is as follows: knives (3/5, 60%), hooks (0/5, 0%), working Area (0/5, 0%), butcher hands (2/5, 40%), nasal swabs (4/5, 80%), rectal swabs (3/5, 60%), chicken (7/8, 88%), mutton (3/5, 60%), and beef (5/7, 71.%).

**TABLE 5:** *coa* and *spa* genes patterns of the 50 MRSA strains isolated from different sources.

				*spa* X region band patterns			*coa* gene band patterns	
Source	Total number of isolates (n)	Isolate names	*spa* + ve isolates	100 bp	200 bp	*coa* + ve isolates	680 bp	891 bp
Knives	5	A1, A13, A28, A35, A40	A1	–	1	A1, A13, A35	–	3
Hooks	5	B1, B2, B3, B4, B5	−ve	−ve	−ve	−ve	−ve	−ve
Working Area	5	C1, C3, C12, C27, C39	−ve	−ve	−ve	−ve	−ve	−ve
Butcher hands	5	D1, D4, D9, D20, D37	D20	–	1	D9, D20	–	2
Nasal swab	5	E1, E2, E6, E11, E14	E1, E2	–	2	E1, E2, E11, E14	–	4
Rectal swab	5	F2, F13, F18, F19, F20	F13, F18	–	2	F13, F18, F20	–	3
Chicken	8	G1, G3, G10, G15, G20, G23, G24, G27	G3, G10, G15, G20, G23	5	–	G1, G3, G10, G15, G20, G23, G27	7	–
Mutton	5	H3, H9, H15, H16, H30	H3, H9, H15	3	–	H3, H9, H15	3	–
Beef	7	I1, I7, I9, I10, 113, I17, I32	I1, I7, I10, I13	4	–	I1, I7, I10, I13, I17	5	–

## Discussion

The emergence and persistent spread of drug-resistant bacteria has become one of the most daunting problems facing the world today (Hayat et al., 2020). Global antibiotic use in low- and middle-income countries increased by 65% from 2000 to 2015 (Dweba et al., 2019). There is evidence that overuse of antimicrobials in food animals leads to the production of drug-resistant bacterial infections in both animals and humans (Mohsin et al., 2019). Many antimicrobials used in veterinary medicine are also listed in the World Health Organization (WHO) catalog of vitally important antimicrobials in hospital settings (World Health Organization [WHO], 2019). Potential risk of spreading MRSA is through contamination of meat sellers, slaughter house workers and people associated with livestock and poultry.

The present study documented the prevalence of MRSA during 2018–2019 in slaughterhouses and meat shops in Rawalpindi-Islamabad, Pakistan. After phenotypic detection, 150 samples of a total of 300 samples were positive for *S. aureus*, with an overall prevalence of 50%. For confirmation of MRSA all 150 isolates were subjected to a disk diffusion test and further molecular analysis. Ninety-five out of one hundred fifty *S. aureus* isolates were considered MRSA by antimicrobial resistance pattern based on the frequency of resistance to cefoxitin (30 μg) which is known as a potential marker for detecting methicillin resistance (Fernandes et al., 2005; Jain et al., 2008; Pourmand et al., 2014). Similar results have been reported in other studies in China, Sudan, United Kingdom, Greece and Algeria (Sergelidis et al., 2015; Ibrahim et al., 2017; Chaalal et al., 2018; Wu et al., 2018; Anjum et al., 2019). All *S. aureus* strains isolated in the current study showed complete resistance to the neomycin, methicillin, ciprofloxacin and tetracycline. Such results are consistent with studies performed in Ethiopia and Nigeria, which document related patterns in neomycin, methicillin and tetracycline resistance (Iroha et al., 2011; Adugna et al., 2013). A similar study by Igbinosa et al. (2016) in Nigeria in 2016 showed 100% resistance of *S. aureus* isolates to methicillin. Resistance to novobiocin, amoxycillin, erythromycin, gentamycin and vancomycin was 52, 70, 76, 82, and 80%, respectively. A similar research conducted in Egypt revealed 78% of *S. aureus* isolates showed resistance to vancomycin and erythromycin, and 100% of the strains showed resistance to gentamycin (Osman et al., 2016). There are many studies in which *S. aureus* antibiotic-resistant strains have been identified in different food products (Hanson et al., 2011; Basanisi et al., 2017; Rong et al., 2017; Haskell et al., 2018; Pekana and Green, 2018; Wu et al., 2018).

The molecular confirmation of *S. aureus* and detection of methicillin resistant *S. aureus* (MRSA) were conducted via *PCR*. This technique has been used previously for the detection of *S. aureus* and methicillin resistant *S. aureus* (Vossenkuhl et al., 2014; Osman et al., 2016; Vaiyapuri et al., 2019). In this study, it was used for detection of *16S rRNA* gene that is specific for bacterial identification, *nuc* gene that confirms *Staphylococcus aureus* presence, *mecA* gene that codes for *protein binding protein 2a (PBP2a)* and is used for the detection of MRSA and for two virulence factors (*spa* gene and *coa* gene) of highly pathogenic *S. aureus*. All 150 isolates were detected positive for *nuc* and*16S rRNA* genes. Of the 150 Staphylococci phenotypically identified in our study only 118 (63%) *S. aureus* isolates were considered MRSA by molecular detection of *mecA* gene.

The *mecA* gene is highly conserved among staphylococcal strains and is being used as a potential biomarker for the detection of methicillin resistance *S. aureus* (MRSA) (Jain et al., 2008). In our study, MRSA prevalence based on *mecA* identification is highest in chicken (23/30, 77%) followed by beef (25/40, 63%), mutton (15/30, 50%), knives (18/40, 45%), nasal swabs (7/20, 35%), working area (11/40, 28%), rectal swabs (5/20, 25%), hooks (7/40, 18%), and butcher hands (7/40, 18%). The highest prevalence of MRSA was found in meat samples in our study, which is comparable to a study conducted in Georgia (Jackson et al., 2013) and higher than those reported in Egypt and Netherland, United States, Korea, and Canada, which showed a 14.5 and 2.5, 1.9, 0.5, and 24.8% prevalence, respectively, in meat samples (Diederen et al., 2007; Kim et al., 2015; Ge et al., 2017; Al-Amery et al., 2019). This high prevalence (79%) of MRSA in the current study may be the result of variations in sample size, sampling sites and raw meat samples from open markets processed through butcher tools and are treated by humans who may be a possible carrier of *staphylococci* isolates. Possible reasons for lower prevalence in some countries may be the sampling season and time, i.e., the samples were obtained in the winter season within 8 h of slaughter and in the early afternoon in order to reduce the risk of contamination. Total of 23 isolates that were susceptible to cefoxitin phenotypically showed presence of *mecA* gene, which is a unique property of these isolates that has not been reported so far in research involving detection of methicillin resistant *S. aureus*.

Our current study findings demonstrated a congruence between phenotypic resistance and molecular typing. Methicillin resistance was identified in 95 isolates checked with cefoxitin disk diffusion technique, while 118 isolates had *mecA* gene. Generally speaking, our results are consistent with studies that indicated the detection of *staphylococci* strains which were *mecA* positive but susceptible to cefoxitin (Martineau et al., 2000; Lee et al., 2004; Osman et al., 2016). The possible explanation is that the resistance pattern of the phenotypic expression can differ depending on the temperature or osmolarity of the media used. It would probably make MRS susceptibility testing by conventional laboratory procedures difficult.

Fifty *mecA* positive samples out of one hundred eighteen were selected for *spa* and *coa* genes typing. The *spa* gene coding for the outer coat protein known as Protein A which is conserved between *S. aureus* strains (Okorie-Kanu et al., 2020). This gene provides sufficient short sequence repeat region (known as the X-region) containing variable number tandem repeats (VNTRs) that are genetically heterogeneous and are used as a single-locus sequence typing target (SLST), commonly known as *spa* typing (Koreen et al., 2004). Protein A an antiphagocytic protein bound to the cell wall with its C-terminal end, the amino terminal end being free outside and binding with the Fc region of IgG (Rathore et al., 2012). In the present study, 18 of the 50 MRSA isolates were identified as positive for the *spa* gene by amplifying the X-region of protein A generating amplicons of two different sizes, 100 and 200 bp. The 200 bp was the most common band in knives, butcher hands, nasal and rectal swab samples, while the 100 bp band was prevalent in meat samples (Chicken, Beef and Mutton). A similar study conducted in Egypt showed *spa* gene segment size ranges from 100, 200, 280, and 290 bp after PCR amplification of MRSA isolates (Salem-Bekhit et al., 2010). A related studies conducted in India showed *spa* gene amplicon sizes after PCR in MRSA isolates using same primer set as (206, 243, 262, 277, 292, 306, and 339 bp), (280, 250, 240, 200, 190, 180, 170, 150, and 140 bp), and in Italy (253 bp), respectively (Casagrande Proietti et al., 2010; Khichar et al., 2014; Bhati et al., 2016). In the current study, MRSA strains in which the *spa* gene is absent, it is proposed that either the *spa* mutation has occurred or the *spa* gene appears to have been absent from these strains. Similarly, previous studies also identified *S. aureus* isolates without *spa* gene (Baum et al., 2009; Momtaz et al., 2010; Salem-Bekhit et al., 2010; Shakeri et al., 2010).

The amplification of the coagulase gene was regarded as a fast and precise method for typing *S. aureus*. Coagulase enzyme is a major virulent element that is secreted by all strains of *S. aureus*. Coagulase trigger the coagulation of plasma at the host and is identification marker for *S. aureus* infection (Himabindu et al., 2009). The heterogeneity among different strains of *S. aureus* is based on the region containing the 81 bp tandem repeats the 3′ coding region of the coagulase gene, which varies in the number of tandem repeats as well as in the position of the *AluI* and *HaeII* restriction sites between the different isolates (Afrough et al., 2013; Javid et al., 2018). In the present study, 27 of the 50 MRSA isolates were found to be positive for coa (Coagulase) gene producing segments of two different sizes, 680 and 891 bp. The 680 bp was the most common band in knives, butcher hands, nasal and rectal swab samples, while the 891 bp was prevalent in meat samples (Chicken, Beef and Mutton). A related studies conducted in India and United Kingdom showed coa gene amplicon sizes after PCR in MRSA isolates ranges from 510–1,000 bp using same primer sets (Hookey et al., 1998; Himabindu et al., 2009; Khichar et al., 2014; Javid et al., 2018).

This research added to the literature by contrasting the phenotypic and molecular characteristics of *S. aureus* in slaughter houses is and discovering the possible transmission modes of MRSA. However, this analysis also has possible drawbacks which cannot be overlooked. First of all, we found the methicillin resistant strains of *S. aureus* via the detection of a *mecA* gene and did not detect a novel *mecA* homolog *mecC* gene, which should be detected in future studies. Also there are no current surveillance programs which allow us to recommend a large scale of research to be carried out to cover the whole country, specifying a large number of sample sizes to be included for the majority of meat consumed in Pakistan.

## Conclusion

The current study concludes that raw meat in the twin cities of Pakistan was contaminated with pathogenic methicillin-resistant *S. aureus* strains that were also resistant to clinically important antimicrobials, which is alarming for public health. This study is the first in Pakistan to report on the detection of *nuc*, *mecA*, *coa*, and *spa* genes positive MRSA. The findings of the present study will significantly add to existing knowledge of veterinary health research as well as food safety by providing proper education and training to slaughterhouse staff that would lead to the low *S. aureus* contamination by butchers, particularly in developing countries around the world. Furthermore the antibiotic resistance rates observed in the current study would highlight the importance of implementation of strict policies and strategies on the prudent use of antibiotics by the public as well as the farming sector. *S. aureus* in the current study were screened for two virulent genes (*coa* and *spa*), thus further in-depth genetic analysis covering the entire country is required including the detection of resistant genes (*blaZ, tetA, tetM, tetK, ermA, ermB, ermC, femA*, etc.), enterotoxins, pvl (*Sea, Seb, Sec, Sed, See*), *SCCmec* types (*SCCmecI-SCCmecIII, SCCmecIva, SCCmecIVb, SCCmecIVc, SCCmecIVd, SCCmecV*), *spa* typing and MLST (Multilocus sequence typing). There is also a need for continuous tracking, and the introduction of improved management methods inside the food chain to reduce contamination of food with MRSA and the eventual spread of the bacteria.

## Data Availability Statement

All datasets generated for this study are included in the article/Supplementary Material and nucleotide sequencing data is submitted in GenBank.

## Author Contributions

AS and JK contributed to the idea or design of the research. JK compiled the data. AS wrote down the first draft of the manuscript and wrote the subsequent revisions of the manuscript. All authors contributed to the article and approved the submitted version.

## Conflict of Interest

The authors declare that the research was conducted in the absence of any commercial or financial relationships that could be construed as a potential conflict of interest.

## Supplementary Material

Supplementary Figure 1 Growth of *S. aureus* on mannitol salt agar. Growth of *Staphylococcus aureus* showing yellow colonies and mannitol fermentation on mannitol salt agar medium.

Supplementary Figure 2 Growth of *S. aureus* on blood agar. *Staphylococcus aureus* showed beta hemolysis on blood agar medium.

Supplementary Figure 3 Gram staining test for the identification of *S. aureus*. Clusters of gram positive cocci observed under microscope.

Supplementary Figure 4 Catalase test for the identification of *S. aureus*. Formation of bubbles after colony mixing with H_2_O_2_ shows catalase Positive result.

Supplementary Figure 5 Coagulase test for the identification of *S. aureus.* Formation of clumps after colony mixing with blood shows coagulase positive result.

Supplementary Figure 6 DNase test for the identification of *S. aureus*. Clear zone around colonies shows DNase positive result after treating with 1 N HCL.

Supplementary Figure 7 Results of antibiotic Resistance tests. Mueller-Hinton agar plates were seeded with *Staphylococcus aureus*. Ten antibiotic disks were placed on each plate. All plates were incubated at 37°C overnight. The diameter of each zone was measured in millimeters and evaluated for resistance using the comparative standard method.

Supplementary Figure 8 Extracted genomic DNA. The extracted DNA for Sample#1–9 was analyzed through gel electrophoresis using 1% gel.

Supplementary Figure 9 Detection of 16S rRNA gene in *S. aureus* isolates: A band (886 bp) corresponding the amplified region of 16S rRNA. First lane = Negative control, 2nd lane = 100 bp ladder, Lane 17 = Positive control, Lane# 3–16 = PCR products of 16S rRNA gene from different sources (Knives, Hooks, Working area, Butcher hands, Nasal swabs, Rectal swabs, Beef, Chicken, and Mutton).

Supplementary Figure 10 Detection of *nuc* gene in *S. aureus* isolates: A band (270 bp) corresponding to amplified region of *nuc* gene. First lane = 100 bp ladder, 2nd Lane = Positive control (*S. aureus* ATCC29213), Lane 16th = Negative control, Lane# 3–15 = PCR products of *nuc* gene from different sources (Knives, Hooks, Working area, Butcher hands, Nasal swabs, Rectal swabs, Beef, Chicken, and Mutton).
