# Supplementary figures and images for: Methicillin-Resistant Staphylococcus aureus (MRSA) in Slaughter Houses and Meat Shops in Capital Territory of Pakistan During 2018–2019
Source: Front Microbiol. 2020 Sep 28;11:577707. doi: 10.3389/fmicb.2020.577707 (PMC7550752; doi:10.3389/fmicb.2020.577707)

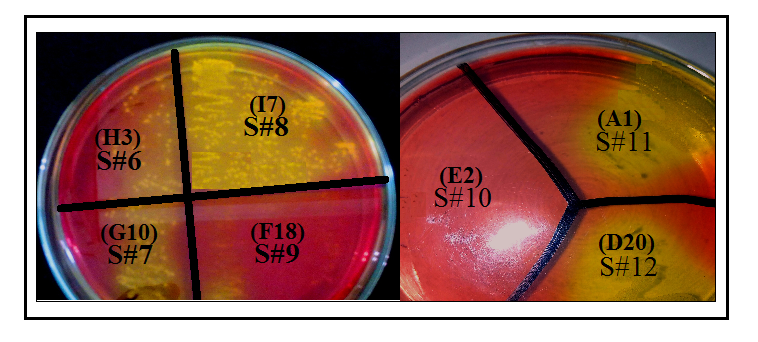

Supplement: Supplementary Figure 1 — Growth of S. aureus on mannitol salt agar. Growth of Staphylococcus aureus showing yellow colonies and mannitol fermentation on mannitol salt agar medium. [file Image_1.TIF]

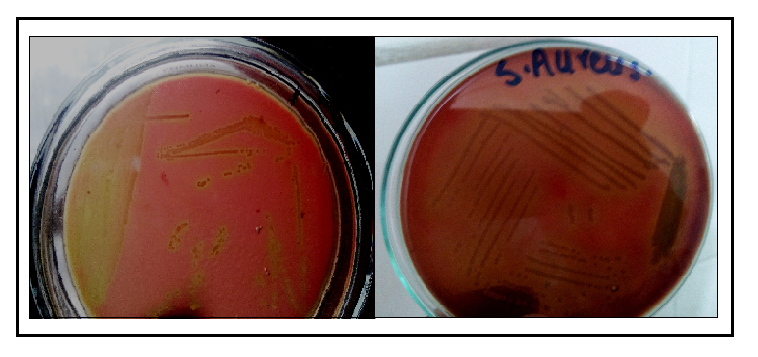

Supplement: Supplementary Figure 2 — Growth of S. aureus on blood agar. Staphylococcus aureus showed beta hemolysis on blood agar medium. [file Image_2.TIF]

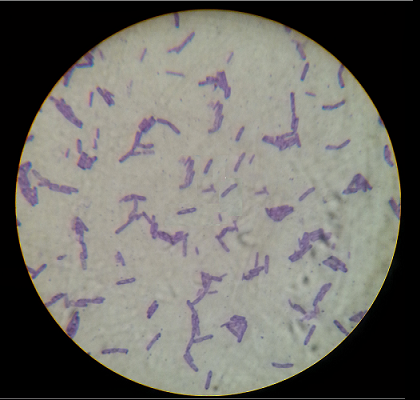

Supplement: Supplementary Figure 3 — Gram staining test for the identification of S. aureus. Clusters of gram positive cocci observed under microscope. [file Image_3.TIF]

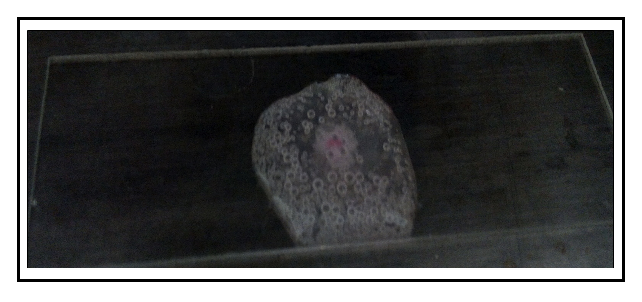

Supplement: Supplementary Figure 4 — Catalase test for the identification of S. aureus. Formation of bubbles after colony mixing with H2O2 shows catalase Positive result. [file Image_4.TIF]

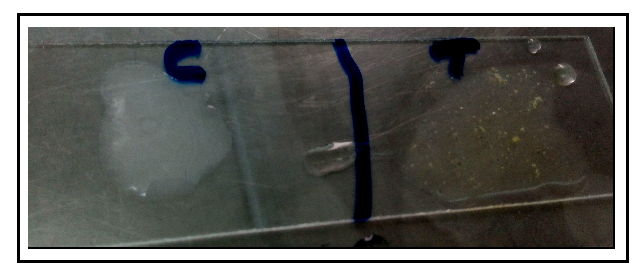

Supplement: Supplementary Figure 5 — Coagulase test for the identification of S. aureus. Formation of clumps after colony mixing with blood shows coagulase positive result. [file Image_5.TIF]

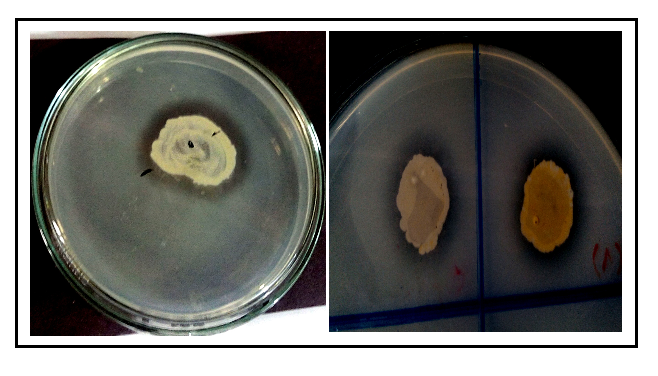

Supplement: Supplementary Figure 6 — DNase test for the identification of S. aureus. Clear zone around colonies shows DNase positive result after treating with 1 N HCL. [file Image_6.TIF]

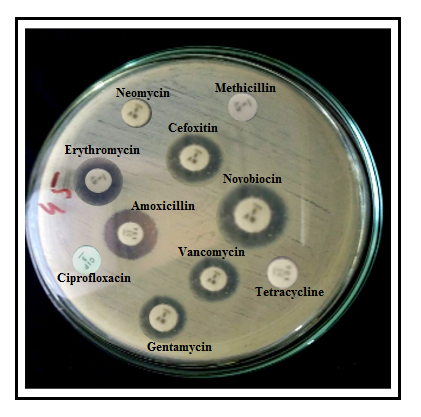

Supplement: Supplementary Figure 7 — Results of antibiotic Resistance tests. Mueller-Hinton agar plates were seeded with Staphylococcus aureus. Ten antibiotic disks were placed on each plate. All plates were incubated at 37°C overnight. The diameter of each zone was measured in millimeters and evaluated for resistance using the comparative standard method. [file Image_7.TIF]

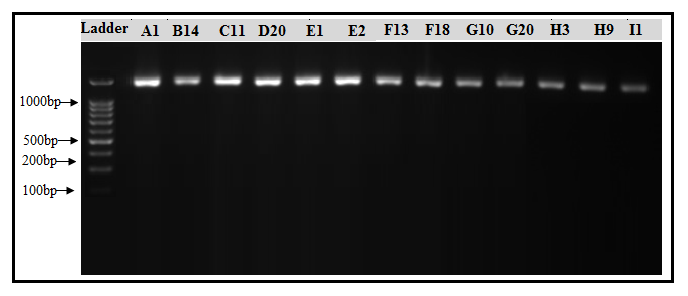

Supplement: Supplementary Figure 8 — Extracted genomic DNA. The extracted DNA for Sample#1–9 was analyzed through gel electrophoresis using 1% gel. [file Image_8.TIF]

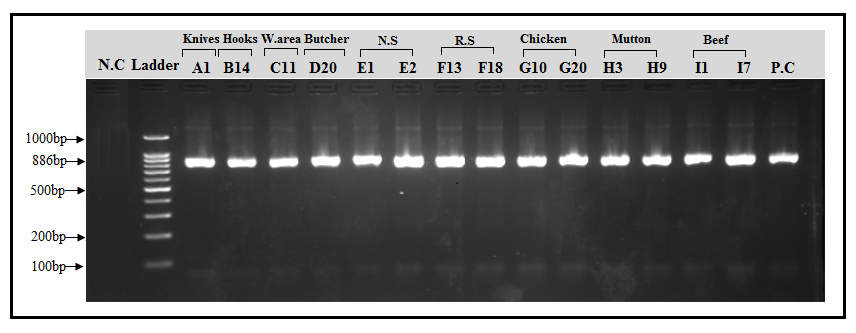

Supplement: Supplementary Figure 9 — Detection of 16S rRNA gene in S. aureus isolates: A band (886 bp) corresponding the amplified region of 16S rRNA. First lane = Negative control, 2nd lane = 100 bp ladder, Lane 17 = Positive control, Lane# 3–16 = PCR products of 16S rRNA gene from different sources (Knives, Hooks, Working area, Butcher hands, Nasal swabs, Rectal swabs, Beef, Chicken, and Mutton). [file Image_9.TIF]

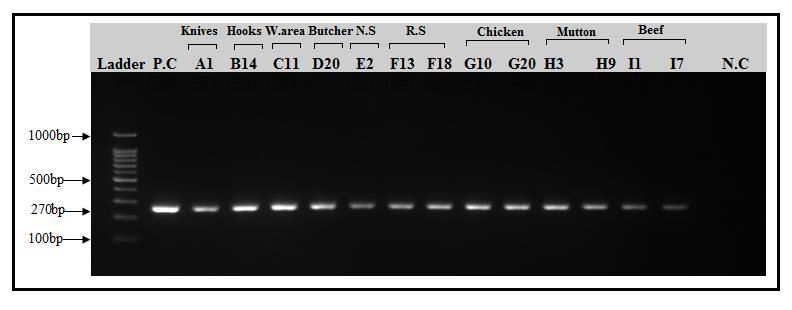

Supplement: Supplementary Figure 10 — Detection of nuc gene in S. aureus isolates: A band (270 bp) corresponding to amplified region of nuc gene. First lane = 100 bp ladder, 2nd Lane = Positive control (S. aureus ATCC29213), Lane 16th = Negative control, Lane# 3–15 = PCR products of nuc gene from different sources (Knives, Hooks, Working area, Butcher hands, Nasal swabs, Rectal swabs, Beef, Chicken, and Mutton). [file Image_10.TIF]
